# Integrated analysis of microRNA and mRNA expression profiles in rats with selenium deficiency and identification of associated miRNA-mRNA network

**DOI:** 10.1038/s41598-018-24826-w

**Published:** 2018-04-26

**Authors:** Yanjing Feng, Yunjie Xing, Zhongwei Liu, Guang Yang, Xiaolin Niu, Dengfeng Gao

**Affiliations:** 1 Department of Cardiology, The Second Affiliated Hospital, Xi’an Jiaotong University School of Medical, Xi’an, Shaanxi P.R. China; 2 Department of Cardiology, Meishan Branch of the Third Affiliated Hospital, Yanan University School of Medical, Meishan, Sichuan P.R. China

## Abstract

Selenium deficiency is closely related with various type of cardiovascular disease. However, the miRNA-mRNA regulatory network in Selenium deficiency related cardiac change remains to be understand. In the present study, a reliable Selenium deficiency rat model was established and confirmed by pathological and biochemical examination. The mRNA and miRNA expression profiles were conducted by microarray technology. Gene Ontology (GO) Analysis and Kyoto Encyclopedia of Genes and Genomes (KEGG) Pathway Analysis was performed to investigate the function of targeted genes, and the relationship between miRNA and mRNA was studied by network analysis. A total of 4931 mRNAs and 119 miRNAs was differentially expressed between any two groups (control group, low-selenium group and selenium supplementation group). GO and KEGG pathway analysis of selected miRNAs target genes found that selenium deficiency was related to several different biological processes. Furthermore, a miRNA-mRNA regulatory network was conducted to illustrate the interaction of miRNAs and these targeted genes. In conclusion, our present study provides a new insight that potential molecular mechanism of Selenium deficiency was a multiply miRNAs and mRNA caused biological change.

## Introduction

Selenium is an essential trace element in mammals. It plays important role in antioxidant, immune system, free radical scavenging and a series of crucial biological process^1^. Thus, selenium deficiency is associated with various type of diseases, especially cardiovascular disease including Keshan disease, atherosclerosis, coronary heart disease, myocardial hemorrhage and necrosis^2–5^. At present, the molecular mechanism of Selenium deficiency related cardiovascular disease remains relatively poorly defined.

The microRNAs (miRNA) are a group of endogenously expressed, non-coding RNA, which function as silencers of several target genes. Prior studies that have noted the importance of miRNAs, which are involved in numerous of biological processes, such as cell proliferation, differentiation, apoptosis and tumorigenesis^6^. Related bioinformatics analysis has shown that 30% of all human gene sets were regulated by around miRNAs^7^. A number of miRNAs have been identified to be involved in cardiac cell survival and death, such as miR-155, miR-874 and miR-200a-5p^8–10^. Our previous work also revealed that the heart failure of the rat with selenium deficiency was probably related to five upregulated miRNAs (miR-374, miR-16, miR-199a-5p, miR-195 and miR-30e) and three downregulated miRNAs (miR-3571, miR-675 and miR-450a)^11^. As has been extensively studied, the expression of miRNAs and their targeted genes are changed in different situations. However, questions have been raised about the miRNA-mediated mRNA regulation. Thus, an Integrated analysis of miRNA and mRNA expression make it possible to construct miRNA-mRNA interactive network, which help us further explore the relationship between cardiovascular disease induced by selenium deficiency and miRNA regulation.

In this study, high-throughput gene chip technology has been used to explore the differential expression of miRNAs and mRNAs and performed a profiling of miRNA expression in selenium deficient rats. Then a comprehensive analysis was conducted for the first time to evaluate the functional miRNA-mRNA interactive network of selenium deficiency. By integrated analysis of miRNA and mRNA expression profiles, our study elucidated the potential biological and molecular mechanism of cardiac dysfunction in selenium deficient rat models.

## Materials and Methods

### Animals

A total of 60 male Sprague-Dawley rats were purchased from the Animal Experimental Center, Xi’an Jiaotong university (Xi’an, China). The average weight was 75 ± 10 g. All rats were raised in the following conditions: 12-hour light/dark cycle, 24–26 °C, 65 ± 4% humidity and free access to water and food. The standard diet (containing 0.2 mg selenium/kg food) was produced by the Animal Experimental Center of Xi’an Jiaotong University and the low-selenium diet (<0.02 mg selenium/kg food) was produced by Trophic Animal Feed High-tech Co. (Jiangsu, China) according to the AIN-93 M formula. All experiments in this study were approved by the Committee on the Ethics of Animal Experiments of Xi’an Jiaotong University and carried out in strict accordance with the recommendations in the Guide for the Care and Use of Laboratory Animals of the National Institutes of Health. Rats were anaesthetized by intraperitoneal injection with 10% chloral hydrate (300 mg/kg body weight) in this work.

### Group and treatment

All the SD rats were randomly divided into three groups: Control (n = 20), low selenium (LS) (n = 20) and selenium supplementation (SS) (n = 20). For the control group, animals were fed with standard diet for 14 weeks and were treated by intraperitoneal injection of physiological saline every day for 21 days. Both of the LS group and SS group, animals were fed with low-selenium diet for 14 weeks, while rats in the LS group were treated by intraperitoneal injection of physiological saline for every day 21 days, rats in the SS group were treated by intraperitoneal injection of sodium selenite (0.05 mg/kg bodyweight; Sigma-Aldrich, St. Louis, MO, USA) for every day 21 days^12^. All the animals were monitored every two days by observing their mental status and activities.

### Selenium concentration detection

The selenium concentration was assessed by the flameless atomic absorption spectrophotometry method using a Z-5000 spectrophotometer (Hitachi, Ltd., Tokyo, Japan) with a cathode lamp of Se (resonance line, 196.0 nm; Photron, Victoria, Australia). Standard selenium solution was used to calibrate the results.

### Histology

The hearts were removed and rinsed in phosphate-buffered saline, fixed in 4% paraformaldehyde for 24 h, embedded in paraffin and cross-sectioned into 10-µm slices. Tissue sections were stained with hematoxylin/eosin and observed by light microscopy.

### Microarray analysis of miRNA and mRNA

The gene microarray analysis of the 3 groups was completed, total RNA from each sample was extracted by using Trizol reagents (Invitrogen) and the miRNeasy mini kit (Qiagen). RNA quality and quantity was measured by using nanodrop spectrophotometer (ND-1000, Nanodrop Technologies, Wilmington, DE, USA) and RNA integrity was determined by gel electrophoresis. the miRCURY™ Hy3™/Hy5™ Power labeling kit (Exiqon, Vedbaek, Denmark) was used according to the manufacturer’s guideline for miRNA labeling. After purification with the miRNeasy mini kit (Qiagen), the Hy3TM-labeled samples were hybridized on the miRCURYTM LNA Array (v.16.0) (Exiqon) according to the array instructions. total RNA from each sample was linearly amplified and labeled with Cy3-UTP. The labeled cRNAs were purified by RNeasy Mini Kit (Qiagen). The labeled cRNAs were hybridized onto the Whole Genome Oligo Array (4 × 44 K, Agilent Technologies). Microarray was then washed by Wash buffer kit (Exiqon) and scanned by the Axon GenePix 4000B microarray scanner (Axon Instruments, Foster City, CA). the GenePix Pro 6.0 software (Axon Instruments) was utilized to extract the data.

### Differentially expressed miRNAs and mRNAs definition

Expressed data was normalized by using the Median normalization method. In this study, fold change (FC) method was utilized to identify differential mRNA and miRNA between these three groups, with following criteria: (1) Fold Change >2 or Fold Change <0.5; and (2) FDR <0.05.

### Series Test of Cluster

Series Test of Cluster (STC) algorithm was utilized to analyze the gene expression dynamics and observe the changes of gene expression under different situations^13,14^. The raw expression values were converted into log2ratio. Using a strategy for clustering the different expression tendencies of differentially expressed mRNAs and miRNAs in each group, we established 8 unique expression profiles, respectively. The expression model profiles were related to the actual or the expected number of genes assigned to each model profile. Significant profiles have higher probability than expected by Fisher’s exact test and multiple comparison test.

### Prediction of miRNA-targeted Gene and construction of miRNA-mRNA network

The miRNA target prediction tools, TargetScan and miRnada, were utilized to further explore the targeted mRNAs, which were regulated by differentially expressed miRNAs^15^. Then We selected mRNAs from the two expected profiles, namely profile NO. 1 and profile NO. 6. These genes closely correlate with the results of regulatory gene expression, including activation and inhibition in the context of heart damage induced by Selenium deficiency. Furthermore, combining the differentially expressed miRNAs and mRNAs as well as the predicted targets for these miRNAs, a core miRNA-mRNA regulatory network was constructed by using the Cytocape software. In the miRNA-mRNA network, the circle represents mRNA and the shape of arrow represents miRNA, and their relationship was represented by one edge. The center of the network was represented by degree. Degree means the contribution one miRNA to the genes around or the contribution one gene to the miRNAs around. The key miRNA and mRNA in the network always have the biggest degrees.

### GO Analysis and KEGG pathway Analysis

GO analysis was applied to analyze the main function of the specific genes with significant differences in the representative profiles of miRNA target genes. Fisher’s exact test was applied to determine the significant GO categories and P values were corrected by FDR. Only GOs that had a P-value of < 0.001 and an FDR of < 0.05 were chosen. Enrichment provides a measure of the significance of the function: as the enrichment increases, the corresponding function is more specific, which helps us to find those GOs with more concrete function description in the experiment.

Pathway analysis was used to find out the significant pathway of the differential genes. Pathway annotations of Microarray genes were download from KEGG (http://www.genome.jp/kegg/). Fisher exact test was also used to find the significant enrichment pathway. The resulting P values were adjusted using the BH FDR algorithm^16^. Pathway categories with *p*-value < 0.05 were reported.

**Figure 1:**
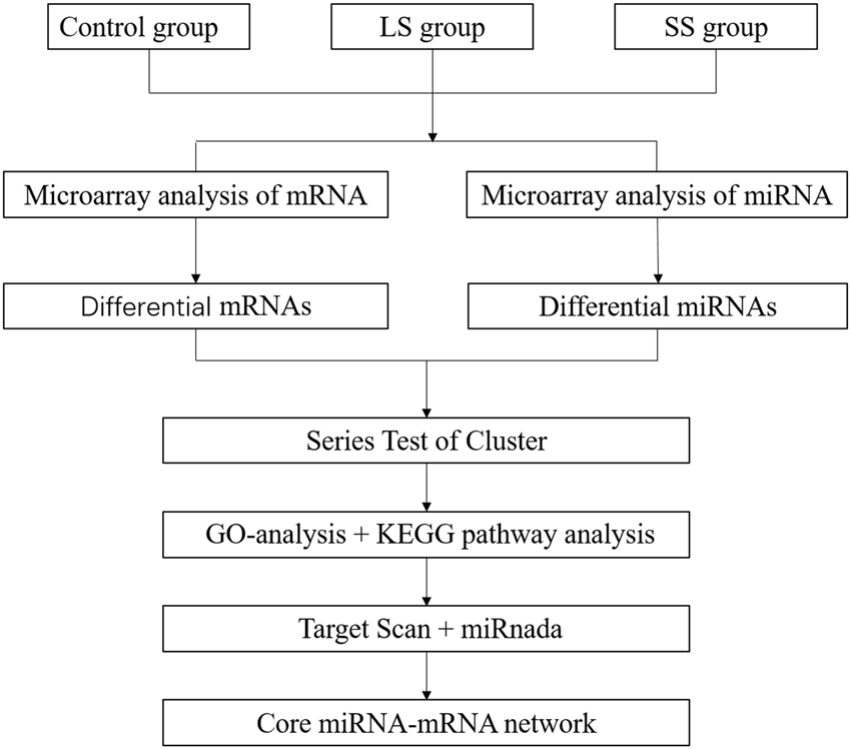
Flow chart of constructing the miRNA-mRNA network associated with selenium deficiency. LS, low selenium; SS, selenium supplementation; GO-analysis, Gene Ontology analysis; KEGG pathway analysis, Kyoto Encyclopedia of Genes and Genomes pathway analysis.

Procedures of constructing the miRNA-mRNA network associated with selenium deficiency were shown in Fig. 1.

### Statistical analysis

All the results were expressed as mean ± standard deviation. Statistical analysis was performed with one-way analysis of variance for multiple comparisons. All the statistical analyses were performed using the software SPSS 18.0 for Windows (PASW Statistics, SPSS Inc., Chicago, IL). P < 0.05 was considered to indicate a statistically significant difference.

## Results

### Selenium concentration in blood samples

**Figure 2:**
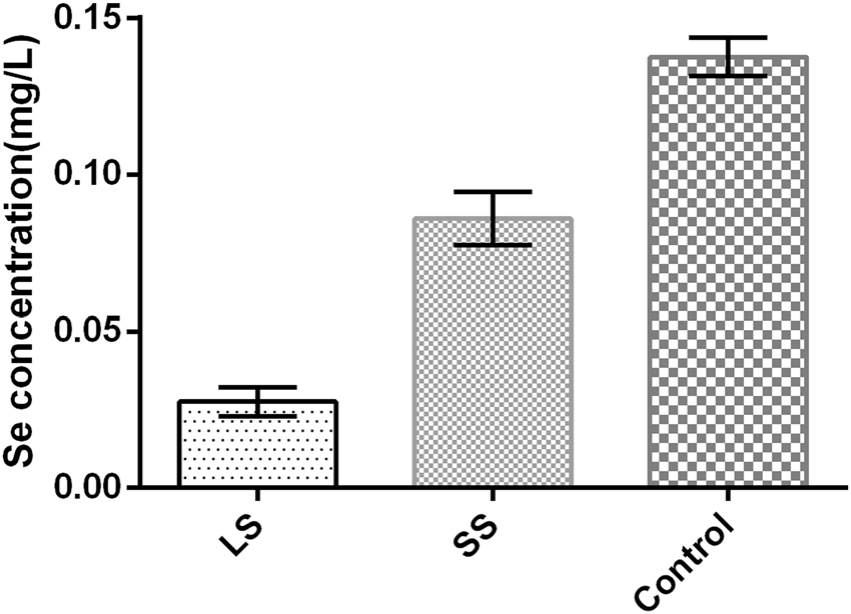
Se concentration for each group. Se concentrations in the blood of rats were analyzed by spectrophotometry. ^*^P < 0.05 vs. control; ^#^P < 0.05 vs. LS. LS, low selenium; SS, selenium supplementation.

The Se concentration in blood was demonstrated in Fig. 2. As shown, compared with the control group, the Se concentration decreased significantly in the LS and SS groups (P < 0.05). After Se supplementation by intraperitoneal injection, Se concentration in blood in the SS group was increased in contrast with LS group (P < 0.05).

### Histological characterization of the heart

**Figure 3:**
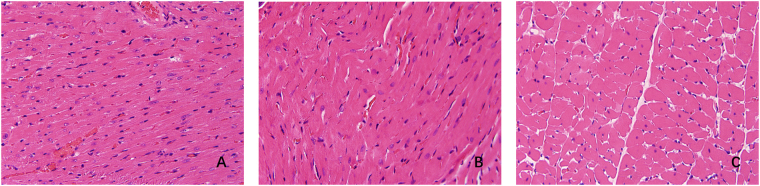
Histological changes of the rat hearts. (**A**) Control, (**B**) LS and (**C**) SS groups. (**A**) Normal structure of myocardial tissue (magnification: x400). (**B**) Swelling and disorganized muscle fibers (magnification, x400). (**C**) Mild swelling and orderly arrangement myocardial fibers (magnification, x400). LS, low selenium; SS, selenium supplementation.

HE staining revealed that histological changes of the rat hearts caused by selenium deficiency (Fig. 3). In the control group, well-connected intercalated discs and normally arranged filaments were seen clearly. Swelling and disorganized muscle fibers were shown in the LS group. Compared with the control group, the structure of myocardial tissue was mildly swelling in the SS group, but their arrangements were ordered relatively.

### Microarray analysis and Significant Differential Gene Analysis

**Figure 4:**
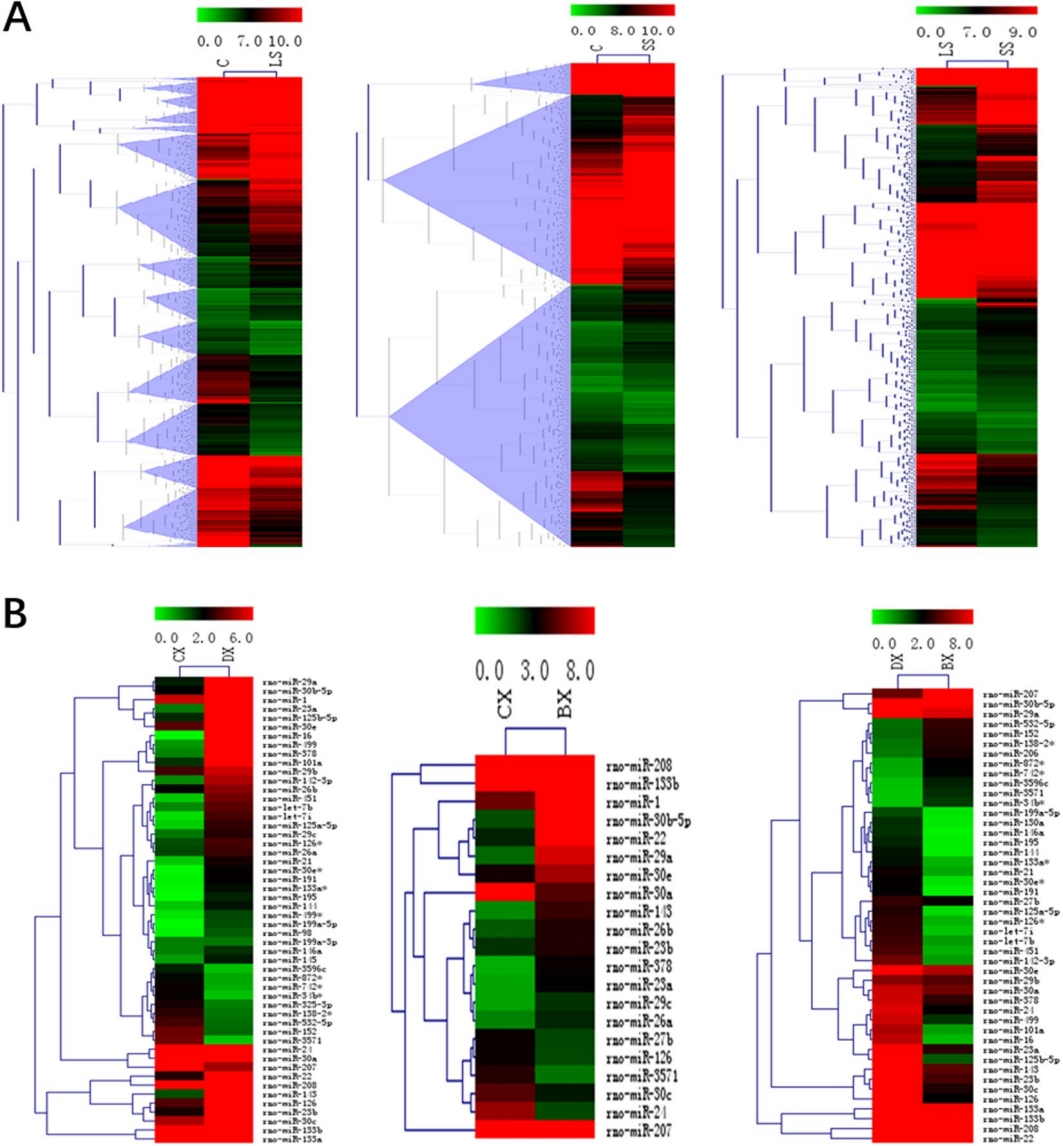
Differential mRNA (**A**) and miRNA (**B**) expression between any two groups of control group, low selenium group and selenium supplementation group.

To identify the possible gene expressional change in selenium deficiency rat models, mRNA and miRNA microarray was performed by using the Affymetrix probe dataset. A total of 4931 mRNAs and 119 miRNAs was differentially expressed with more than 2.0-fold change between any two groups, as shown in heat map analysis (Fig. 4). Of 4931 differentially expressed mRNAs, the expression of 2514 genes were upregulated and 2417 genes were downregulated. Of 119 differentially expressed miRNAs, the expression of 65 miRNAs were upregulated and 54 miRNAs were downregulated.

### Series Test of Cluster of miRNA and mRNA expression profiles

**Figure 5:**
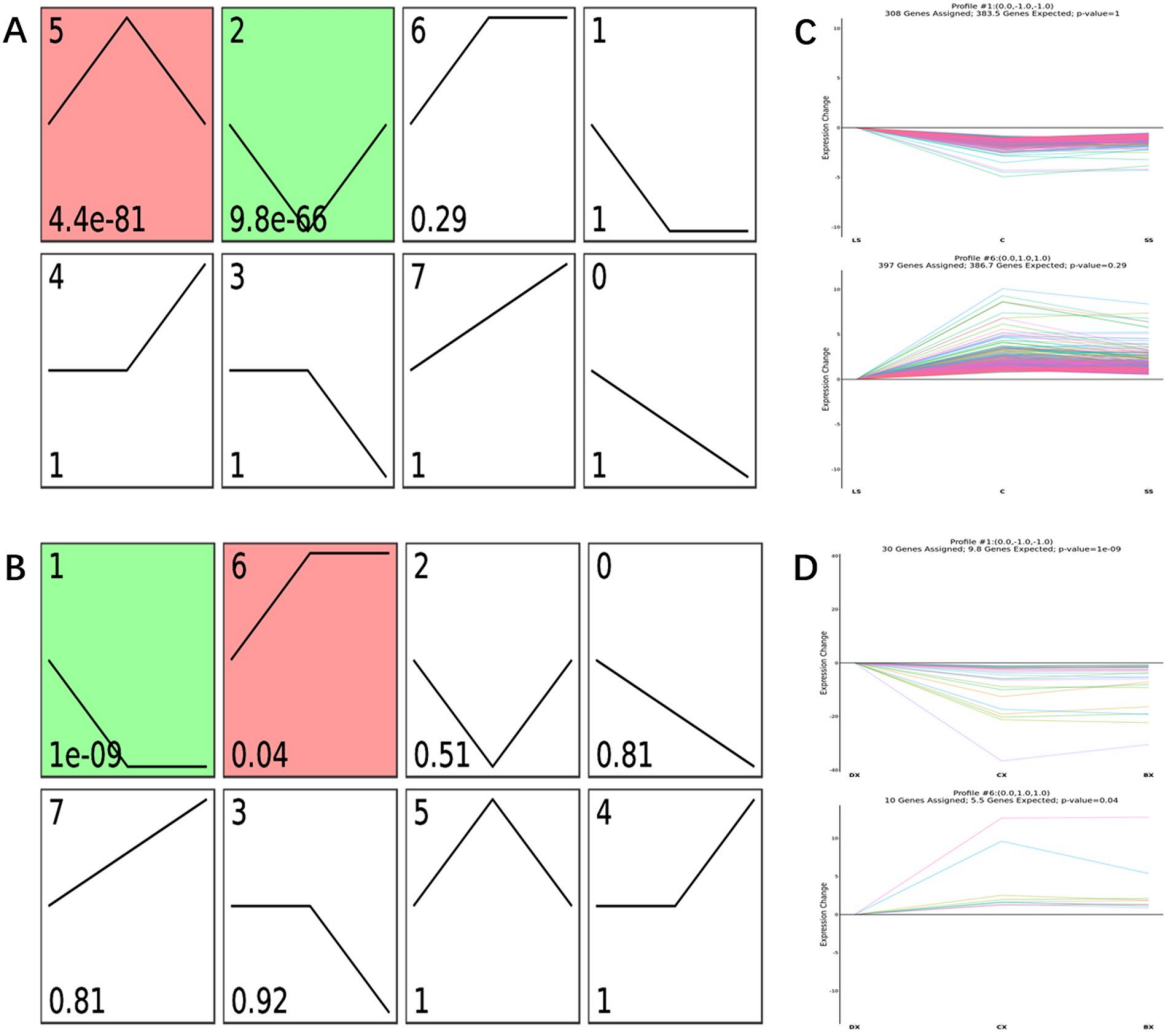
STC analysis of differentially expressed miRNA and mRNA in microarray. (**A,B**) Eight model profiles were utilized to illustrated the expression patterns of 4931 differentially expressed mRNA (**A**) and 119 expression expressed miRNA transcript (**B**), respectively. Each box represents a different model expression profile. The p value and corresponding model profile number were listed in upper and lower corner of each profile box, respectively. (**C,D**) 2 model expression profiles of mRNA transcripts (**C**) with statistically significant (p < 0.05) and relevant expression profiles miRNA (**D**) are shown, respectively. Model expression profile number, the number of genes assigned to its corresponding profile, the number of genes expected to its corresponding profile and p-value are shown on the top of each profile. The horizontal and vertical axes represent indicated time points and the time series of gene expression levels after Log normalized transformation, respectively. Fisher exact test for (**A** and **B**).

To further characterize mRNA and miRNA expression profiles, we establish 8 expression profiles for the 119 differentially expressed miRNAs and 4931 mRNAs by using STC method in STEM software. Each profile indicates an expression model including a set of miRNAs or mRNAs with analogous expression pattern, respectively. As shown in Figs 5, 2 expression patterns of mRNA (Profile NO. 2 and NO. 5) and 2 expression patterns of miRNA (Profile NO. 1 and NO. 6) showed statistically significance (p < 0.05), respectively. Among these patterns, we chosen the 2 significant miRNA expression patterns, which were accorded with the pattern that we expected. As we known, miRNA leading to downregulation of target gene expression at transcriptional or translational levels^17^, A negative relationship between miRNAs and their targeted genes has been reported in the literature. Then relevant mRNA expression profiles (Profile NO. 1 and NO. 6) were also chosen to further analysis. Profile NO. 1 contained 30 miRNAs and 308 mRNAs whose expression rapidly declined from LS group to control group but stable at SS group. This group of miRNA might be involved in the heart pathological changes of selenium deficiency. while Profile NO. 6 contained 10 miRNAs and 397 mRNAs whose expression remained temporally stable from SS group to control group but decreased at SS group. To the 30 miRNAs of profile NO. 1, we found 36738 target genes, to the 10 miRNAs of profile NO. 6, we found 9612 target genes, respectively.

### GO Analysis and KEGG pathway Analysis of putative target genes

**Figure 6:**
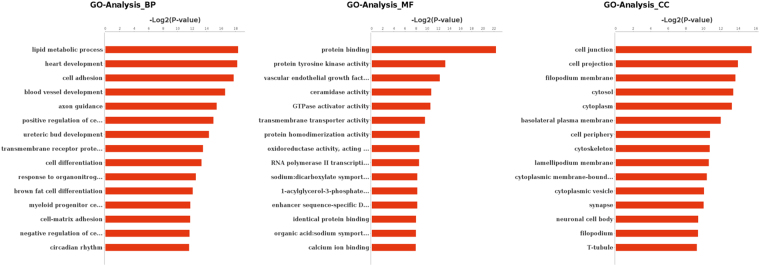
GO analysis for targeted genes of differentially expressed miRNA. −Log2 (p value) of the corresponding biological process(BP), molecular function(MF) and cellular component(CC) (the top 15 GO annotations of each section). Log2 (P-value) is the negative logarithm of P-value; bigger Log2 (P-value) indicates smaller P-value.

**Figure 7:**
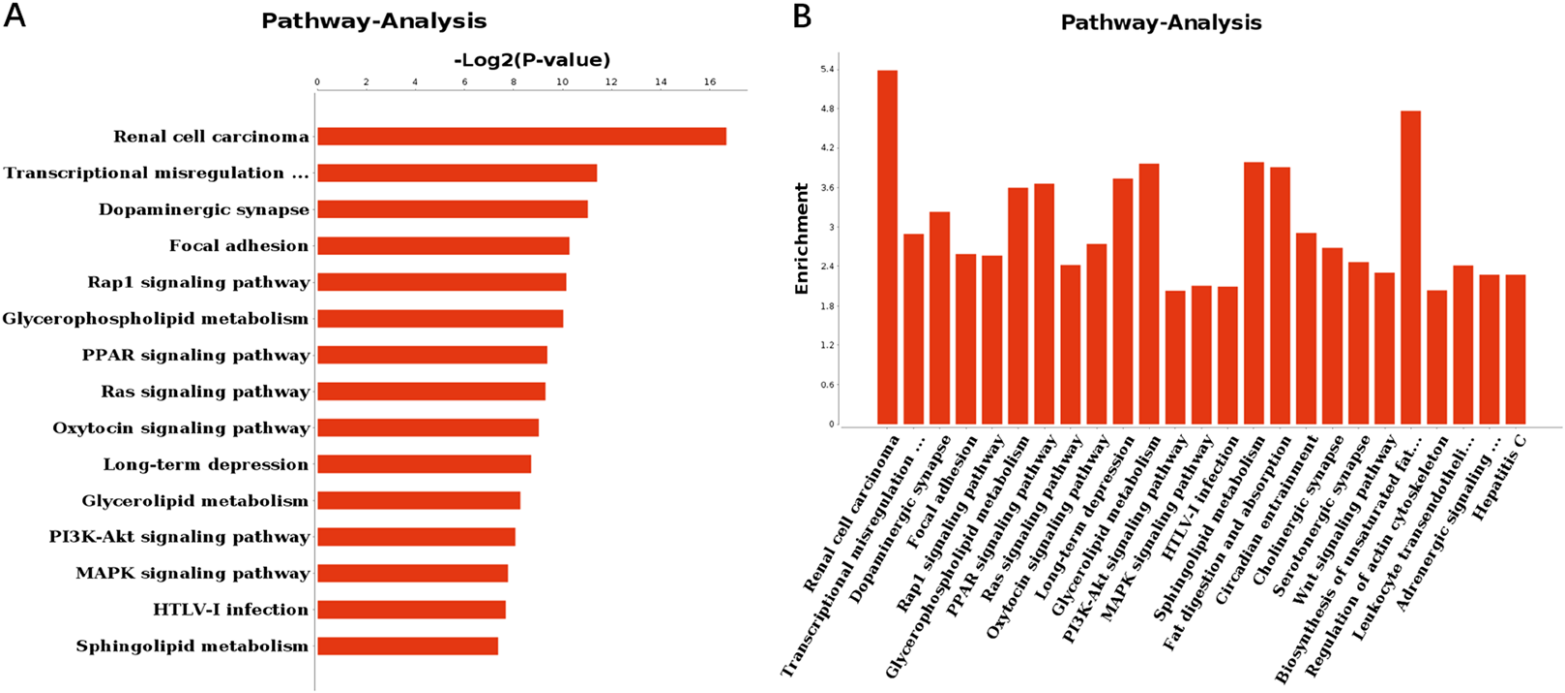
Pathway analysis for targeted genes of differentially expressed miRNA. −Log2 (p value) of the corresponding pathway (**A**). Enrichment for the top 25 selected pathway (**B**).

With the aim of exploring the relationship between differentially expressed genes and Selenium deficiency induced heart damage, GO and KEGG pathway analysis were applied respectively. GO analysis of representative profiles of miRNA target genes were shown in Fig. 6. on the biological process level, GO terms were mainly related to metabolism and development associated process, such as lipid metabolic process and heart development. On the molecular function level, GO terms were mainly involved in protein binding and enzyme activity. On the cellular component level, GO terms were mainly included cell junction, cell projection and cytoplasm. KEGG Pathway analysis evaluating the enriched pathways for the representative profiles of miRNA targeted genes showed that some of them were involved in signal transduction pathways (Fig. 7), such as focal adhesion kinase, Rap1 signaling pathway, PPAR signaling pathway, Ras signaling pathway and Wnt signaling pathway.

### Analysis of the miRNA-mRNA network

**Figure 8:**
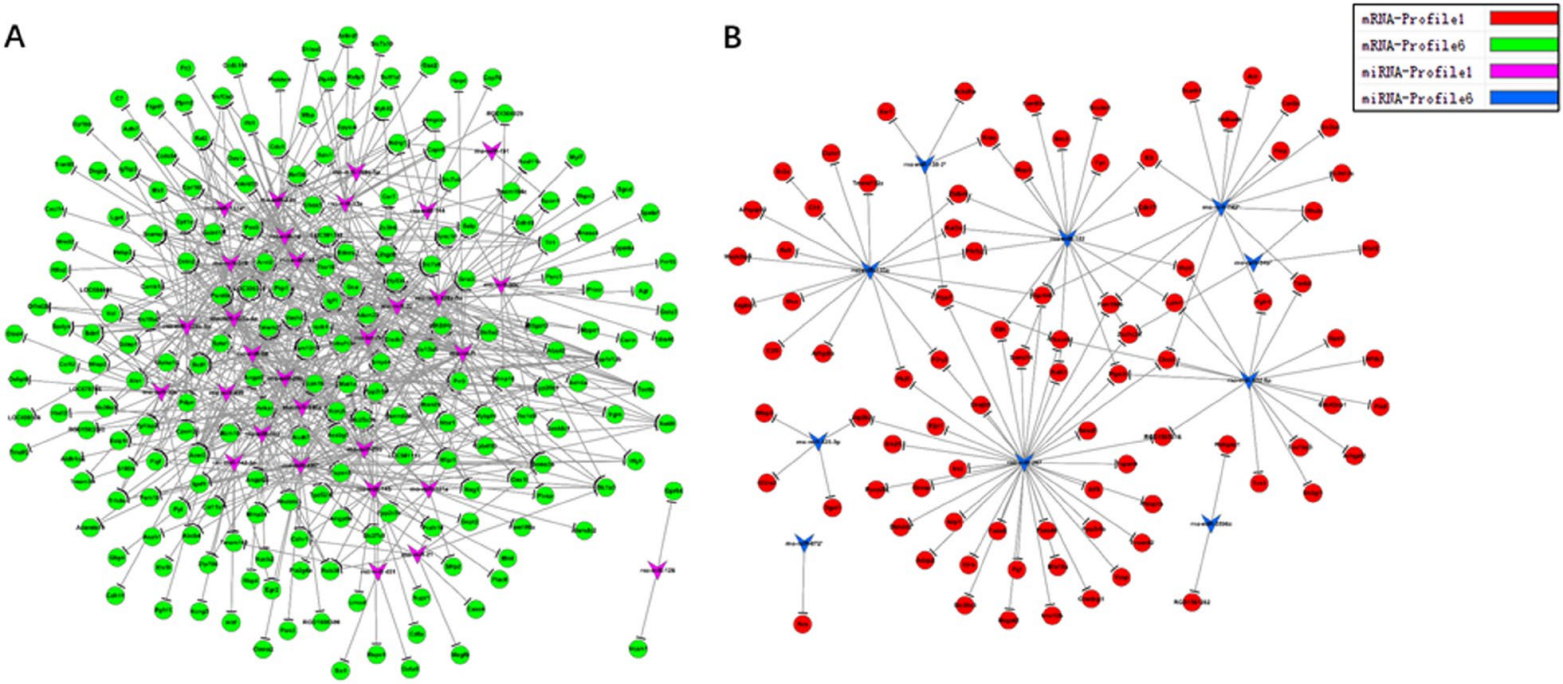
The miRNA-Gene-Network for miRNAs in the Profile NO. 1 (**A**)/Profile NO. 6 (**B**) and their target mRNA, the circle represents mRNA and the shape of arrow represents miRNA, and their relationship was represented by grey edge.

Depend on these miRNAs (in the Profile NO. 1 and Profile NO. 6) and their targeted genes, the miRNA-mRNA network was created to summarize the interaction of miRNAs and these targeted genes. The highest degrees always mean the key miRNA in the network. As shown in Fig. 8A, 30 downregulated miRNAs which were belonged to Profile NO. 1 were in the miRNA-mRNA network. While these miRNAs which had higher degree were in the center of the network, such as rno-miR-125a-5p is the one that regulates the most genes, as many as 41 predicted target genes. These decreased miRNAs may lead to the increased expression of their target genes, for instance, Tspan5, Lgr4 and Mmd2, to regulate related signal transduction. The network of 10 upregulated miRNAs of Profile NO. 6 was illustrated in Fig. 8B, including rno-mir-207, rno-mir-152, rno-mir-133a, may repress the expression of negative modulators, for example, Fads6, Samd14 and Fbxo46, respectively.

To summarize all the results, we have displayed that these crucial miRNAs may function as important regulators by lipid metabolic process, heart development, protein binding, enzyme activity, cell junction, cell projection and signal transduction, thus playing pivotal roles in heart damage caused by selenium deficiency.

## Discussion

As one of the essential trace elements of mammalian, selenium regulate various biological processes such as redox balance, immunity, aging and disease^18,19^. Selenium-deficiency can cause a variety of cardiovascular diseases, most of them are induced by oxidative damage and inflammation^20^. At present, the molecular mechanism of Selenium-deficiency remains to be understood. Due to being incorporated as the unique amino-acid selenocysteine into selenoproteins, selenium is essential for the synthesis, metabolism and function of hormones^1,21^. Therefore, a comprehensive understanding of the specific role of selenium in cardiovascular disease is meaning for the treat strategy. The integrated analysis of miRNA-mRNA regulatory network was utilized to help us improve the cognition of gene regulations on selenium deficiency related cardiac disease.

Of all the differentially expressed genes, a substantial proportion were related with lipid metabolic process, heart develop, cell adhesion, blood vessel development, cell differentiation, signal transduction and positive regulation of cardiac muscle hypertrophy. These differentially expressed mRNAs might take part in the selenium deficiency mediated biological process. We also acquired the associated signal pathways which were modulated by the differentially expressed mRNAs through KEGG analysis. Among the 44 pathways with p value < 0.05, 4 pathways were related to metabolism of Lipid, including glycerophospholipid metabolism, glycerolipid metabolism, sphingolipid metabolism and fatty acid metabolism. Since selenoproteins adjust apolipoprotein E levels and regulate the gene expression of cholesterol synthesis, metabolism, and transport, they are critical to lipoprotein metabolism^22^. Selenium deficiency caused cellular responses often lead to the induction of inflammation and oxidative overload involving multiple signaling pathways^10,23^. Several signaling pathways were mediated by the differentially expressed mRNAs, such as focal adhesion, Rap1 signaling pathway, PPAR signaling pathway, Ras signaling pathway, Oxytocin signaling pathway, MAPK signaling pathway and Wnt signaling pathway. As mentioned in the literature, Selenium deficiency can support oxidation of two oxidation sensors and activate Wnt pathway^24^. Combining the function and pathway analysis for differentially expressed genes in selenium deficiency rat model, we may have concluded that cardiac cell might develop different cellular responses and gain corresponding pathological changes due to deficiency of selenium.

Pertaining to the different miRNAs, we successfully identified 30 downregulated miRNAs and 10 upregulated miRNAs in our rat models. Of these miRNAs, such as rno-miR-16, rno-miR-199a-5p, rno-miR-195, rno-miR-30e, which had been reported to be involved in selenium deficiency induced heart failure^11^. Although several research teams have conducted the clinical research to clarify the relationship between selenium deficiency and cardiovascular disease^25–28^, few studies have paid attention to the important roles of miRNAs and miRNA-mRNA interaction of molecular mechanism.

The integrated analysis of the miRNA-mRNA regulatory network was a reliable strategy to enhance the understanding of gene regulation. Specific miRNA-mRNA interactions and high-throughput analyses of the downstream effects of miRNAs on mRNAs have been the focus of many research studies^29,30^, which provided valuable insights on regulation of gene expression. As to Network-based prediction were extensively applicated in various cancer to diagnosis, predict tumor clinical phenotypes and test personalized drug targets^31–34^. We can also develop an algorithm to predict these diseases which are associated with selenium deficiency at early stage, by using machine learning method and selenium deficiency related miRNA-mRNA network. Actually, selenium deficiency caused cardiovascular disease is a complex process, several potential mechanisms such as oxidative stress, inflammation have been validated^35,36^. However, the molecular mechanism of miRNAs regulates gene (mRNA) expression of selenium deficiency has not been studied. It is necessary to identify the target with opposite expression patterns of miRNA and select the pivotal miRNA as well as their target mRNAs in selenium deficiency related cell change. With the help of bioinformatics analysis, we filtered several miRNAs which were in the center of the miRNA-mRNA regulatory network and corresponding target genes, such as rno-miR-125a-5p. In addition, more work need to be conducted to reveal the concrete regulatory mechanism of selenium deficiency related cardiovascular disease.

In conclusion, this report reveals that a number of miRNAs and mRNAs that correlated with selenium deficiency caused cardiovascular disease, and also illustrated a core functional miRNA-mRNA network which was meaningful for reveal the regulation from miRNAs to mRNAs in the selenium deficient rat model.
